# Alterations in knee biomechanics and motor performance following 3 months training with the Football+ and 11+ warm-up programs among amateur female players. A three-armed cluster allocated, non-randomized intervention study

**DOI:** 10.1016/j.jesf.2026.200463

**Published:** 2026-03-14

**Authors:** Mojtaba Asgari, Martin Hägglund, Benedikt Terschluse, Maximilian Sueck, Kevin Nolte, Marcus Schmidt, Thomas Jaitner

**Affiliations:** a Training and Movement Science Group, Institute for Sport and Sport Science, TU Dortmund University, Dortmund, Germany; b Department of Health, Medicine and Caring Sciences, Unit of Physiotherapy, Linköping University, Linköping, Sweden; c Sports Scientist/Strength and Conditioning Coach Girls' and Women's Football Department, BVB09, Dortmund, Germany

**Keywords:** Women's football, Injury prevention, Knee biomchenics, The Football+ program

## Abstract

**Objective:**

This cluster alocated comparative study (DRKS00036644) primarily evaluated the effects of the Football+ and the established FIFA 11+ (the 11+) on knee biomechanical risk factors. A secondary outcome included performance measures and their retention following a 10-week no-intervention period.

**Methods:**

Three German amateur women's teams (24.3 ± 5.3 years, 1.73 ± 0.07 m, 64.3 ± 8.0 kg) completed the introductory sessions and were team allocated into the Football+ (n = 22), 11+ (n = 19), or control (n = 16) groups. Baseline assessment included a standardized 3D motion analysis of knee biomechanics during single-leg landing and cutting maneuvers and a performance test battery (sprinting, counter movement jump (CMJ), agility, and dribbling speed. Following a 3-month, twice-weekly supervised intervention, post-intervention testing was performed. A 10-week follow-up tested retention of the performance outcomes. Statistical significance was set at α ≤ 0.05.

**Results:**

ANOVA revealed significant time and time × group interactions across multiple knee biomechanical variables. The Football+ showed consistently larger magnitudes of change compared with the 11+, while the control group demonstrated limited changes (p = .005-0.047; η^2^ = 0.14-0.24). Similar interaction effects were observed for sprinting, agility, and dribbling performance (p = .002-0.01; η^2^ = 0.13-0.23). Performance improvements were not retained after a 10-week no-intervention period.

**Conclusion:**

The Football+ and 11+ programs improved high-risk movement patterns associated with an increased risk of knee injury, with more consistent and larger effects observed for the Football+. Improvements in performance measures were observed only following the Football+, supporting its potential as a time-efficient, dual-purpose warm-up. The performance improvements were not maintained following the no-intervention period.

## 1 Introduction

Warm-up strategies as the final preparatory stage before intensive physical activity, are expected to serve dual purposes: reducing injury risk and enhancing motor performance.^1^ However, most injury prevention programs (IPPs), including the established FIFA 11+ (the 11+), designed in the form of warm-up programs have demonstrated limited effectiveness in performance enhancement 2, 3, 4. One argumentative explanation for this issue is that majority of IPPs, including the 11+, have been developed primarily by medical professionals with a focus on injury risk reduction and limited input from sport scientists, resulting in programs that emphasize prevention but insufficiently target performance.^5^ This major limitation combined with implementation challenges such as time constraints, lack of perceived relevance to football-specific goals, and low engagement and enjoyment of the players have restricted the real-world adoption of the 11+ and other IPPs, despite their well-proven efficacy on injury prevention (IP).^6^, ^7^, ^8^, ^9^

Although consistent implementation of the 11+ has shown to reduce the incidence of lower limb, overuse, acute, lower back, and knee injuries from 30 to 63% 10, 11, 12, 13, 14, 15 low real-world adaptation rates in Germany, Australia, and Saudi Arabia ranging from only 10 to 40%^8^^,^^9^^,^^16^ indicate that even strong evidence for IP alone may not be sufficient to promote widespread adoption of IPPs. A dual-focus strategy that integrates established IP principles with performance-oriented training science may offer a more aligned and adoptable IP approach.

The Football+ was developed as a dual-purpose warm-up program aiming to simultaneously modify biomechanical risk indicators and improve football-specific motor performance. Building on the existing literature and recommendations to improve the 11 + ^2^^,^^5^^,^^7^^,^^8^^,^^17^, ^18^, ^19^, ^20^, ^21^, the Football+ integrates intensive football-specific exercises with proven IP methods into a structured, engaging, and time-efficient warm-up. It features three progressive modules: (1) dynamic mobility, strength, and neuromuscular control; (2) small-sided games to enhance coordination, decision-making, and technical skills; and (3) plyometric, sprint, and agility drills for explosive movement development. A three-level progression system enables players to gradually increase training intensity based on their readiness and feedback.

Previous investigations revealed that the Football+ yields superior acute benefits in sprinting, agility, jumping, and dribbling speed compared to the 11+.^22^ Further, it has demonstrated significant improvements in landing technique among female players with and without a history of severe ankle or knee injuries, again compared to the 11+ and control group.^23^ Meanwhile, the program's effects on injury risk factors as well as long-term performance adaptations remain unexplored. With regard to conceptual models from “the sequence of prevention” (van Mechelen et al., 1992) to Team-sport Injury Prevention (TIP) cycle (O'Brien et al., 2018), a critical factor for all IPPs is the need to understand and assess their effects on injury risk factors.^24^^,^^25^

Knee kinematic and kinetic parameters are key indicators of knee injury risk, particularly for ACL injuries 26, 27, 28. A detailed biomechanical analysis is thus essential to evaluate the clinical relevance and effectiveness of the Football+ program in mitigating knee injury risk. In addition, evidence on the 11+ impacts on knee biomechanics remains limited and inconsistent 29, 30, 31. For instance, Arundale et al. (2021) found no significant improvements in dynamic knee valgus during landing tasks among amateur female players following a season-long application of the program.^30^ Conversely, Dix et al. (2021) reported a clinically meaningful reduction in knee valgus during a 90-degree cutting maneuver after 12 weeks of implementation in a similar cohort.^29^ The inconclusive nature of the 11+ literature highlights the need for further investigation into the underlying biomechanical mechanisms by which the 11+ may mitigate knee injury risk.

This study, therefore, aimed to evaluate the effects of three months training with the Football+ or the 11+ with a control group (CG) on key knee kinematic and kinetic parameters during high-risk movement patterns, single-leg landing (SLL) and cutting, as well as on performance measures. We hypothesized that the Football+ would demonstrate at least comparable positive effects on knee biomechanics relative to the 11+ and superior outcomes compared to the CG, while yielding greater improvements in performance measures than both the 11+ and CG.

## 2 Methods

### 2.1 Study design and ethics

This three-arm, cluster-allocated, non-randomized interventional study (DRKS00036644) included two intervention groups and one control group (CG). Cluster allocation at the team level was chosen to ensure feasible and standardized implementation of the warm-up interventions within routine training settings; however, the potential risk of selection bias associated with this approach is acknowledged and addressed in the Limitations section. The study design comprised baseline and post-intervention assessments of knee biomechanics and motor performance, as well as a follow-up assessment of performance outcomes after a 10-week no-intervention period. The study was conducted in accordance with the Declaration of Helsinki and received ethical approval from the Ethics Committee of TU Dortmund University (GEKTUDO-2022-43). All participants provided written informed consent prior to enrollement.

### 2.2 Inclusion and exclusion criteria

Eligible participants were female football players aged 18 years or older with at least 3 years of football experience, injury-free at baseline, having 3 training sessions weekly in addition to a weekend match, and registered with their teams for the 2022-2023 season. Exclusion criteria included missing two or more intervention sessions, concurrent participation in other organized sports, a history of severe lower extremity injury 6 weeks before study commencement, and absence from any measurement session.

### 2.3 Participants

A priory power analysis was conducted using G∗Power (version 3.1.9.4) for a repeated-measures ANOVA (within-between interaction, 3 groups × 2 time points). It estimated a sample of 42 as suitable for an expected effect size of 0.25, an α error probability of 0.05 and a power of 0.80.^32^ Participants were recruited from three women's teams competing in German amateur football leagues. Initially, 57 players (24.3 ± 5.3 years, 1.73 ± 0.07 m, 64.3 ± 8.0 kg) enrolled in the study voluntarily, completed on-site instructional workshops and underwent baseline demographic, motor performance, and biomechanical assessments. However, 15 players, primarily from the CG, were excluded due to dropouts or other exclusion criteria, resulting in a final sample of 42 players with complete performance and biomechanical datasets. The larger dropout in the control group was primarily due to scheduling conflicts, sustaining injuries and personal reasons unrelated to the study protocol. Group-specific characteristics were as follows: the Football+ (n = 20; age: 25.5 ± 1.08 years; height: 1.68 ± 0.14 m; body mass: 62.7 ± 2.25 kg), 11+ (n = 16; age: 22.9 ± 1.17 years; height: 1.70 ± 0.08 m; body mass: 65.5 ± 2.05 kg), and CG (n = 6; age: 23.8 ± 2.9 years; height: 1.71 ± 0.03 m; body mass: 66.5 ± 2.7 kg).

### 2.4 Study preparation

During the 2022-2023 preseason, participants attended three familiarization sessions addressing laboratory protocols, interventions, and measurement procedures. Biomechanical assessments were carried out in the biomechanics laboratory at the Institute for Sport and Sport Science, TU Dortmund University. Motor performance assessments were conducted at each team's training facility on artificial turf, in comparable time of day (evenings), and similar weather conditions (cloudy, 50-60% humidity), while temperatures differed accoring to the season (20-23 °C in July, 11-14 °C in October, 2-5 °C in December).

### 2.5 Intervention

Both interventions consist of three parts, lasting 20-25 min in practice and require minimum equipment. The 11+ was implemented according to the original protocol described by Soligard et al..^33^ The Football+ poster and manual are available at https://doi.org/10.17877/DE290R-26479 and https://doi.org/10.17877/DE290R-26478. To support adherence and proper implementation, players received comprehensive educational materials, including video tutorials, program-specific manuals, and posters. Close collaboration with the coaching staff was maintained throughout the intervention period via regular communication from the lead researcher and on-site support from the research assistants who were blinded to the study's hypotheses and outcomes. Each intervention group completed their assigned warm-up twice per week over a 12-week period, starting in week four of the preseason and continuing through week 10 of the competitive season. This was followed by a 10-week no-intervention phase during which the teams discontinued their assigned programs and resumed their traditional warm-up routines. This was intended to determine whether any observed performance gains were directly attributable to the interventions and whether those effects would persist without continued exposure. The CG continued its common warm-up routine throughout the entire study. This typically included light jogging, static stretching, basic technical drills, and 2-3 min of moderate-paced running across the pitch.

### 2.6 Biomechanical data acquisition

To evaluate the programs‘ effects on biomechanical measures a standardized test battery comprising single-leg landing and cutting tasks was administrated in a pre-post design. Following a 5-min standardized warm-up on a treadmill at 7-9 km/h and familiarization trials for both the landing and cutting tasks, players‘ anthropometrics were documented. This was followed by a static standing calibration trial during which the participants stood upright on a force plate with arms extended laterally and feet shoulder-width apart. This position was used for full-body skeletal modelling.^28^^,^^34^

### 2.7 SLL test

Players first performed single-leg drop landings from a 30-cm box onto a force plate. They were instructed to step off and land on the same leg, maintaining balance for at least 3 s post-landing. Trials were repeated until three valid attempts per leg were recorded. A trial was considered valid if the entire foot contacted the force plate and the participant maintained stability without extra steps or visible loss of balance.^28^^,^^34^

### 2.8 Cutting maneuover

Participants then performed cutting maneuvers after a 5-m approach run. They were instructed to execute the cut at match-like intensity, simulating a real 1-vs-1 offensive scenario. The task involved changing direction at approximately 45° angle using a pre-assigned leg, while the cutting angle was not strictly controlled, allowing for natural execution, with the direction and sequence of cuts randomized to avoid bias. A trial was valid if the entire foot landed within the force plate boundaries and no premature deceleration or missteps occurred.^28^^,^^34^ All assessments were supervised by two trained investigators (MA, MS), who monitored technique, ensured protocol compliance, and paused the session in case of fatigue, discomfort, or technical issues.

### 2.9 Motion capturing and data processing

The tasks were captured by a markerless motion capture system including 14 high-speed video cameras (200Hz, Oqus 4, Qualisys, Gothenburg, Sweden) integrated with two AMTI force plates (1000 Hz; Watertown, Massachusetts, USA).^35^^,^^36^ Qualisys Track Manager (QTM) was used to synchronize kinetic and kinematic data in real time, to calibrate the capture volume, and to export trial recordings. Recorded video and force data were subsequently processed using Theia3D (Theia Markerless Inc., Canada).^35^^,^^36^ Initial ground contact was defined as the first frame in which vGRF exceeded 10 N. Data were processed using Visual3D (C-Motion Inc.) The average of three valid trials per limb was used for each test condition.

### 2.10 Kinetic and kinematic measures

Knee valgus angle at initial contact (KValIC), knee valgus moment at initial contact (KValMomIC), maximum knee valgus moment (MaxKValMom), vertical ground reaction force (vGRF), knee flexion angle at initial contact (KFlexIC), and maximum knee flexion angle (MaxKFlex) were calculated as primary indicators of knee injury risk, particularly ACL injuries 26, 27, 28^,^^37^.

### 2.11 Performance assessment

**Fig. 1:**
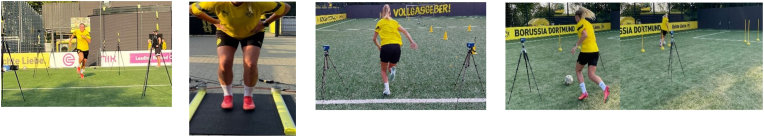
Performance test battery: a. linear sprint, b. countermovement jump, c. illinois agility test, d. dribbling test.

A standardized test battery was administrated at baseline, immediately after the 3-month intervention, and 10 weeks post-intervention to evaluate the programs‘ effects on motor performance and the retention of the potential gains. The test battery included linear 20-m sprint,^3^ countermovement jump (CMJ),^4^ Illinois agility (IA) test,^3^ and dribbling speed (DS) test.^38^ Testing procedures adhered to procedures outlined by Asgari et al.^22^ Dual-light timing gates were used for time-based measurements including sprinting, agility, and dribbling (90-110 cm height). Jump height was recorded via the OptojumpNext system (Microgate, Bolzano, Italy).^39^ To avoid acute warm-up effects from the Football+ or 11+ programs, all teams performed a standardized 10-min general warm-up before testing (see Fig. 1).

### 2.12 Statistical analysis

Descriptive statistics were computed for all outcomes. After confirming normality and homogeneity of variance, a 2 × 3 repeated-measures ANOVA and follow-up post-hoc LSD tests were used to assess within- and between-group differences. Significant time × group interactions from pre-to post-test were interpreted as program effects. Effect sizes were reported as partial eta squared (η^2^) and classified as small (0.01), medium (0.06), or large (0.14).^40^ Statistical analyses were performed using ‘‘R‘‘version 4.5.0, and SPSS version 29, with significance set at α < 0.05.

## 3 Results

Minor deviations from normal distribution were noted for a few variables, including vGRF, and KFlexICL. However, given the moderate sample size (n = 42) and the robustness of repeated measures ANOVA to moderate violations of normality, all primary analyses proceeded using parametric methods.^41^

### 3.1 Compliance and exposure time

Of the 25 scheduled intervention sessions, only one was cancelled due to complete training cancellation, resulting in a compliance rate of 96% in both interventions. Between pre and post tests, total training exposure amounted to 720 h in the Football+ group and 576 h in the 11+ group (lower training hours in the 11+ group refer to the smaller number of players in that group). Of these, 176 and 141 h specifically dedicated to the respective programs (approximately 25% of the total training time).

### 3.2 Program effects on the knee biomechanics

Analysis of the SLL and cutting outcomes revealed side and task-specific effects across the groups. Significant time × group interactions were observed in favor of the Football+ group, especially for KFlexIC, vGRF, and KValIC, while the 11+ group showed moderate improvements and the CG did not change during the study. Details are presented in Table 1, Table 2.

**Table 1:** Pre-post changes in knee biomechanics during the SLL.

variable	limb	group	pre (mean ± sd)	post (mean ± sd)	time effect p	time × group p	effect size (ηp^2^)	Interpretation and post hoc analyses
KFlexIC	L	Football+	16.6 ± 3.7	21.9 ± 6.0	<0.001	0.071	0.308	The Football+ showed the largest within-group effect (d = 0.86), while the 11+ displayed a moderate improvement (d = 0.47).
		11+	15.3 ± 4.0	18.0 ± 4.2				
		Control	18.7 ± 3.6	20.0 ± 7.9				
KFlexIC	R	Football+	18.2 ± 3.4	21.7 ± 3.8	<0.001	0.009	0.310	The Football+ and 11+ showed significant improvments in knee flexion during landing.
		11+	17.8 ± 2.8	19.4 ± 3.3				
		Control	16.2 ± 4.6	16.4 ± 3.6				
KValIC	L	Football+	−1.15 ± 1.91	−0.31 ± 1.24	0.003	0.541	0.210	The Football+ produced the greatest change toward neutral knee alignment. The 11+ showed a mild reduction in valgus angle.
		11+	−0.80 ± 1.56	−0.39 ± 1.59				
		Control	−1.39 ± 2.17	−0.58 ± 3.33				
KValIC	R	Football+	−2.54 ± 2.41	−1.30 ± 1.39	0.003	0.040	0.205	The Football+ significantly improved knee valgus control. The 11+ showed mild effect; while the control group showed no change.
		11+	−1.83 ± 1.45	−1.27 ± 1.02				
		Control	−1.40 ± 1.49	−1.37 ± 1.36				
KValMomIC	L	Football+	0.156 ± 0.170	0.080 ± 0.059	0.184	0.170	0.045	Both the Football+ and the 11+ reduced valgus moment, but changes were not statistically significant.
		11+	0.137 ± 0.127	0.069 ± 0.102				
		Control	0.085 ± 0.139	0.130 ± 0.118				
KValMomIC	R	Football+	0.075 ± 0.138	0.063 ± 0.085	0.590	0.947	0.008	No meaningful change were observed in any group. Valgus moments remained stable.
		11+	0.100 ± 0.110	0.097 ± 0.129				
		Control	0.102 ± 0.108	0.077 ± 0.133				
KValMomMAX	L	Football+	1.030 ± 0.367	0.871 ± 0.285	0.001	0.223	0.239	The Football+ and 11+ reduced peak valgus moments, but no interaction effect was observed.
		11+	1.240 ± 0.454	0.992 ± 0.248				
		Control	0.961 ± 0.335	0.914 ± 0.298				
KValMomMAX	R	Football+	1.001 ± 0.308	0.874 ± 0.280	0.511	0.068	0.011	No meaningful change in the groups. Variables remained stable.
		11+	1.071 ± 0.234	1.028 ± 0.212				
		Control	0.930 ± 0.390	1.027 ± 0.268				
vGRF	L	Football+	3.75 ± 0.67	3.42 ± 0.63	0.006	0.030	0.180	The Football+ showed a significant decrease in impact force, the 11+ showed small improvement while the CG worsened.
		11+	3.98 ± 0.63	3.71 ± 0.52				
		Control	4.01 ± 0.93	4.11 ± 1.24				
vGRF	R	Football+	3.78 ± 0.76	3.56 ± 0.67	<0.001	0.034	0.250	The Football+ significantly reduced vGRF, the 11+ improved slightly, and the CG showed no change.
		11+	4.11 ± 0.69	3.89 ± 0.52				
		Control	4.20 ± 1.26	4.23 ± 1.24				
KFlexMAX	L	Football+	58.4 ± 9.2	57.5 ± 8.2	0.739	0.873	0.003	No meaningful change in the groups. Variables remained stable.
		11+	53.3 ± 9.6	53.6 ± 11.0				
		Control	51.9 ± 8.5	51.3 ± 12.4				
KFlexMAX	R	Football+	60.3 ± 8.6	58.0 ± 7.3	0.462	0.277	0.014	No meaningful change in the groups. Variables remained stable.
		11+	55.8 ± 9.8	55.9 ± 8.1				
		Control	55.2 ± 9.8	55.5 ± 7.7				

KValIC= Knee valgus angle at initial contact, KValMomIC = knee valgus moment at initial contact, MaxKValMom = maximum knee valgus moment, vGRF = vertical ground reaction force, KFlexIC = knee flexion angle at initial contact, MaxKFlex = maximum knee flexion angle.

**Table 2:** Pre-post changes in knee biomechanics during cutting.

variable	limb	group	pre (mean ± sd)	post (mean ± sd)	time effect p	time × group p	effect size (ηp^2^)	Interpretation and post hoc analyses
KFlexIC	L	Football+	20.6 ± 2.9	24.5 ± 3.4	0.004	0.040	0.197	The Football+ and 11+ showed a significant increase in knee flexion, indicating improved shock absorption on the left side.
		11+	17.6 ± 2.7	20.4 ± 4.5				
		Control	24.4 ± 6.4	23.7 ± 3.6				
KFlexIC	R	Football+	22.2 ± 4.7	25.5 ± 5.2	<0.001	0.475	0.300	All groups improved, but with no interaction effect. The Football+ led the improvement, indicating better landing strategies across groups.
		11+	19.7 ± 4.7	22.5 ± 3.3				
		Control	21.4 ± 4.7	22.7 ± 3.9				
KValIC	L	Football+	−3.13 ± 3.2	−1.82 ± 1.7	0.965	0.050	0.143	Football+ showed the most favorable change, with valgus angle moving toward neutral.
		11+	−1.76 ± 1.8	−1.71 ± 1.7				
		Control	−0.79 ± 3.4	−2.09 ± 4.4				
KValIC	R	Football+	−4.48 ± 1.9	−3.00 ± 1.5	0.114	0.032	0.161	Significant group effect: Football+ improved frontal plane control. 11+ showed improvement.
		11+	−3.67 ± 3.6	−2.63 ± 3.2				
		Control	−2.71 ± 3.5	−3.62 ± 3.0				
KValMomIC	L	Football+	−0.18 ± 0.16	−0.07 ± 0.10	0.179	0.013	0.199	The Football+ showed a significant reduction in medial knee loading. 11+ showed no change.
		11+	−0.15 ± 0.12	−0.17 ± 0.16				
		Control	−0.19 ± 0.23	−0.19 ± 0.26				
KValMomIC	R	Football+	−0.26 ± 0.21	−0.13 ± 0.11	0.295	0.047	0.145	The Football+ reduced knee valgus moment with large effect sizes; the 11+ showed minimal change.
		11+	−0.22 ± 0.15	−0.19 ± 0.11				
		Control	−0.29 ± 0.12	−0.35 ± 0.17				
KValMomMAX	L	Football+	0.55 ± 0.32	0.41 ± 0.18	0.036	0.125	0.108	All groups improved over time, especially the Football+. No significant interaction was oberved.
		11+	0.55 ± 0.30	0.45 ± 0.27				
		Control	0.48 ± 0.08	0.52 ± 0.06				
KValMomMAX	R	Football+	0.58 ± 0.23	0.37 ± 0.26	0.341	0.096	0.113	No significant changes were observed.
		11+	0.48 ± 0.53	0.53 ± 0.24				
		Control	0.62 ± 0.40	0.61 ± 0.27				
vGRF	L	Football+	2.14 ± 0.55	1.83 ± 0.38	0.004	0.182	0.193	The Football+ significantly reduced impact forces during landing. The 11+ improved slightly.
		11+	2.34 ± 0.68	2.18 ± 0.52				
		Control	2.53 ± 1.14	2.48 ± 0.73				
vGRF	R	Football+	2.31 ± 0.60	2.11 ± 0.52	0.357	0.019	0.184	Significant group interaction changes favoring the Football+
		11+	2.47 ± 0.60	2.25 ± 0.64				
		Control	2.54 ± 0.93	2.79 ± 0.83				
KFlexMAX	L	Football+	64.3 ± 6.3	67.0 ± 5.5	0.207	0.005	0.240	Significant group difference favoring the Football+.
		11+	63.6 ± 7.3	63.3 ± 7.2				
		Control	72.5 ± 9.5	66.4 ± 8.2				
KFlexMAX	R	Football+	66.3 ± 4.9	67.9 ± 5.0	0.425	0.026	0.171	Group interaction observed: the Football+ and 11+ showed improvement.
		11+	66.5 ± 6.6	67.4 ± 7.3				
		Control	70.4 ± 5.8	66.0 ± 6.1				

KValIC= Knee valgus angle at initial contact, KValMomIC = knee valgus moment at initial contact, MaxKValMom = maximum knee valgus moment, vGRF = vertical ground reaction force, KFlexIC = knee flexion angle at initial contact, MaxKFlex = maximum knee flexion angle.

For the SLL, within-group effect sizes were predominantly moderate-to-large in the Football+ group (d ≈ 0.60-0.90) for primary landing mechanics, whereas effects in the 11+ were small-to-moderate and trivial in the control group. Observed effect sizes for significant interaction effects were moderate-to-large (ηp = 0.18-0.31), indicating meaningful intervention-related changes despite reduced sample size in the control group. Observed power for significant interaction effects ranged between 0.72 and 0.88.

For the cutting task, significant interaction effects were associated with moderate-to-large effect sizes (ηp^2^ = 0.145–0.240), indicating meaningful intervention-related changes in frontal and sagittal plane mechanics during cutting. Within-group effect sizes were consistently larger in the Football+ group compared with the 11+ and control groups.

### 3.3 Program effects on motor performance

**Fig. 2:**
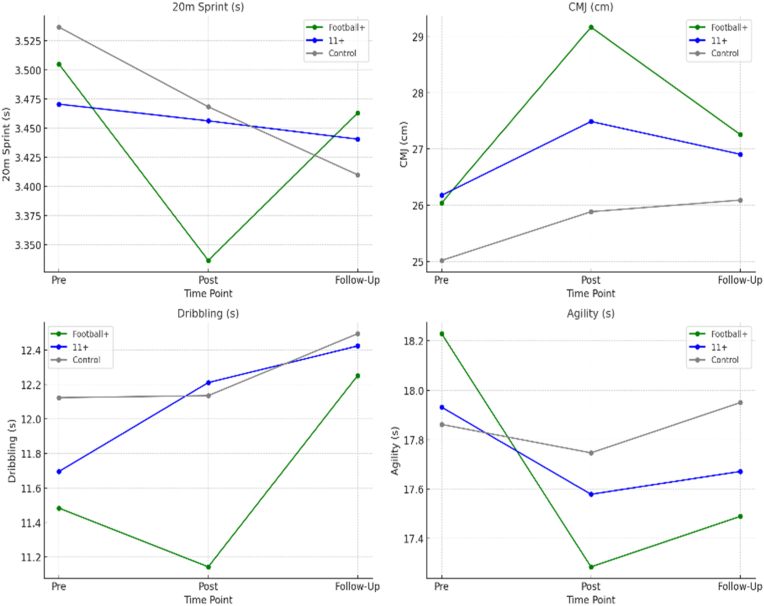
Changes in performance measures at baseline, post-intervention and follow-up testing.

A significant time × group interaction was observed in sprinting, IA, and DS, (p < .05), favoring the Football+ group. According to the post hoc tests, the Football+ showed the most substantial improvements in both sprint (p = .003) and agility (p = .002) times, outperforming the 11+ and control groups. For dribbling, the Football+ group demonstrated meaningful enhancement post-intervention (p= .01), while the 11+ group showed inconsistent or even deteriorating performance (Table 3). Significant improvement in CMJ was observed in both intervention groups again favoring the Football+. However, none of the performance gains were maintained after the 10-week no-intervention period: sprinting (p = 0.23), CMJ (p = 0.47), DS (p = 0.65), IA (p = 0.32) (Fig. 2).

**Table 3:** Pre-post changes in motor performance.

measure	group	pre (mean ± SD)	post (mean ± SD)	follow-up (mean ± SD)	time effect (p)	time × group (p)	effect size (ηp^2^)
20m Sprint	Football+	3.51 ± 0.12	3.34 ± 0.12	3.46 ± 0.13	0.003	0.003	0.142
	11+	3.47 ± 0.13	3.46 ± 0.12	3.44 ± 0.15			
	Control	3.54 ± 0.10	3.47 ± 0.12	3.41 ± 0.10			
CMJ (cm)	Football+	26.04 ± 3.41	29.17 ± 3.05	27.26 ± 2.88	<0.001	0.081 (ns)	0.195
	11+	26.18 ± 2.34	27.49 ± 2.07	26.91 ± 2.42			
	Control	25.02 ± 4.25	25.88 ± 4.10	26.09 ± 2.89			
Dribbling (s)	Football+	11.48 ± 0.59	11.14 ± 0.59	12.25 ± 0.74	<0.001	0.01	0.215
	11+	11.70 ± 1.05	12.21 ± 0.61	12.42 ± 1.09			
	Control	12.12 ± 0.89	12.14 ± 0.97	12.49 ± 0.71			
Agility (s)	Football+	18.23 ± 0.54	17.29 ± 0.44	17.49 ± 0.29	<0.001	0.002	0.230
	11+	17.93 ± 0.86	17.58 ± 0.58	17.67 ± 0.45			
	Control	17.86 ± 0.50	17.75 ± 0.56	17.95 ± 0.60

## 4 Discussion

This study evaluated the effects of the Football+ on key indicators of knee injury risk and performance enhancement in amateur female players compared to the established 11+ and a CG. A supervised adherence rate of 96% in this study played a pivotal role in the successful outcomes of both IPPs, particularly the Football+.^42^ Further, an analysis of training exposure revealed that approximately 25% of total training time was devoted to warm-up activities. This significant investment reinforces the practical need for warm-up routines that simultaneously support both IP and performance enhancement, especially in amateur settings where training time is limited. This may also help explain why compliance with IPPs remains low, despite their well-established preventive benefits. In amateur football, where teams typically train twice weekly, coaches often prioritise tactical, technical, and physical preparation leaving little room for stand-alone IPPs, which is why “lack of time” has frequently been cited as a barrier to implementation.^6^^,^^7^

Overall, our findings suggest that the Football+ program not only improves biomechanical risk factors associated with knee injury risk but also improves important aspects of motor performance, such as sprinting, agility, dribbling, and jumping with large effect sizes. These dual benefits underscore the Football+ as a time-efficient, practical and engaging alternative to traditional IPPs which may potentially gain higher acceptance and fidelity in real practice. Research demonstrates that application of dynamic warm-ups before athletic activity offers physiological, psychological, neurological, and cardiovascular benefits, all of which contribute to enhanced performance and reduced injury risk.^1^

Improvements during SLL were predominantly seen in the dominant leg, whereas cutting performance improvements were largely bilateral, and this may reflect habitual loading patterns and task specificity in football. Players naturally rely more on their dominant limb for kicking, cutting, and stabilizing, which could lead to differential neuromuscular adaptations over time. These side-specific adaptations highlight the importance of including both dominant and non-dominant limb assessments when evaluating intervention efficacy. Clinically, although asymmetries were present, the magnitude of differences were relatively small and likely not detrimental; however, addressing these asymmetries may further optimize performance and reduce injury risk.

It should be noted that our kinematic and kinetic analyses relied on markerless motion capture technology. While this method allows for efficient and non-invasive data collection in field settings, it might be less precise than marker-based systems, particularly in estimating very small joint angles and moments. Consequently, some knee kinetics and kinematics outcomes, especially subtle differences between limbs should be interpreted with caution. Future research combining markerless and gold-standard marker-based systems could further validate these findings.

Increased knee flexion has been consistently associated with a reduction in anterior tibial shear forces and ACL loading, thereby lowering the risk of ACL injury.^43^^,^^44^ This suggests that the Football+ effectively promotes safer landing strategies. This aligns with our previous study, which demonstrated the efficacy of the Football+ in improving landing quality, as measured by the Landing Error Scoring System (LESS), among female players both with and without a history of severe ankle and knee injuries.^23^

In addition, the Football+ group displayed reduced knee valgus angles and moments, a finding of particular importance given that excessive dynamic valgus has been identified as a strong predictor of ACL injury risk, especially among female players 44, 45, 46. The incorporation of cutting and directional-change drills in the Football+ program likely contributed to these improvements by reinforcing proper alignment of the lower extremity complex under dynamic loading conditions. Our findings, on the other hand, echo's Arundale et al. (2021) reporting no significant improvements in dynamic knee valgus during landing tasks among amateur female players following a season-long application of the 11+.

Another key finding was the reduction of vGRF. Elevated vGRFs are known to increase mechanical stress on the knee and hip joints and are often associated with both acute ligament injuries and overuse conditions.^47^ The Football+ likely addressed this through the integration of plyometric training combined with technique-oriented landing tasks, which train players to absorb forces more efficiently through increased knee and hip flexion.

The first and third components of the Football+ include a combination of dynamic stretches, functional strength, and plyometric exercises which are associated with favorable adaptations in landing and cutting mechanics. Strengthening exercises, particularly those targeting the gluteal, hamstring, and core muscle groups have been shown to improve lower limb alignment and reduce dynamic valgus and knee joint loading 48, 49, 50, 51. Similarly, plyometric training has been consistently linked to increased knee flexion at initial contact and decreased ground reaction forces, primarily by improving joint stiffness regulation, proprioception, and neuromuscular control.^49^^,^^52^^,^^53^ These mechanisms align with the observed improvements in knee biomechanics across both SLL and cutting tasks, supporting the program's capacity to target injury-relevant movement patterns effectively.

Although some of these exercises are also included in the 11+ program, the overall dosage of dynamic and plyometric drills in the Football+ is nearly double, likely contributing to its greater impact. In contrast, the 11+ contains a higher proportion of static exercises, particularly in part two^54^ that are more effective in optimizing neuromuscular system and improving proprioception, both of which are essential for reducing the risk of severe ankle and knee injuries.^55^ Such exercises also promote musculoskeletal adaptations, including increased joint range of motion and improved force production.^1^^,^^56^

Although previous studies have reported inconsistent effects of the 11+ on jumping performance, especially among male players, our findings showed notable improvements in jump ability among female participants, which aligns with some earlier studies.^2^ This discrepancy may be explained by sex-specific differences in physical performance, where the moderate intensity of the 11+ may sufficiently challenge female players, but not their male counterparts. In contrast, the Football+ incorporates nearly twice the volume and intensity of plyometric and anaerobic exercises compared to the 11+ likely explaining its superior effects on sprinting, agility, and jumping performance in our cohort.^57^^,^^58^

Dribbling performance, a technical skill closely tied to sport specificity, improved significantly only in the Football+ group, demonstrating that the football-specific components of Football+ resulted in enhanced dribbling speed and coordination, while these effects were not seen in the 11+ group, being a more generic program. These findings are consistent with those of Daneshjoo et al. (2013) And Zarei et al. (2018).^3^^,^^59^ The football-specific drills and small-sided games embedded in the Football+ appear to be critical in enhancing sport-relevant motor skills, supporting existing evidence on the effectiveness of game-based training methods for technical development.^60^

The CG followed a traditional warm-up routine that included light jogging across the pitch, prolonged static stretching, and short intervals of moderate-paced running. While it is often assumed that knowledge transfer into practice is more effective in European amateur women's football, our observations suggest otherwise. Unfortunately, outdated warm-up routines remain prevalent, highlighting the need for improved education and dissemination of evidence-based practices at the grassroots level.

Notably, the performance gains observed in our study were not maintained after the 10-week no intervention period. This underscores the importance of ongoing implementation, aligning with the training principle of reversibility, which states that physiological adaptations diminish without continued stimulus intervention.^61^^,^^62^ Therefore, regular incorporation of the Football+ into weekly training sessions is strongly recommended, though additional research is needed to explore the Football+ impacts on other biomechanical measures, as well as effects on injury incidence. In particular, further comprative studies on the Football+ impacts on groin muscles as well as ankle biomechanics are needed since they account for nearly 12% and 22% of total football injuries at the amateur level.^63^ While the 11+ has shown the lowest effects on these injuries, the Football+ integrates the Coppenhagen exercise and higher dosage of plyometrics to address this gap.

### 4.1 Conclusion and practical implications

A 3-month supervised application of both the Football+ and 11+ programs led to improvements in knee kinematics and kinetics, with the Football+ demonstrating superior efficacy in modifying biomechanical risk indicators in amateur female players. Beyond its injury prevention potential, the Football+ also significantly enhanced dribbling speed, agility, vertical jump, and sprinting performance, whereas the 11+ yielded meaningful improvements only in vertical jump. Given the limited training time available in amateur football, the Football+ appears to offer a time-efficient, engaging, and dual-purpose program that can be seamlessly integrated into routine practice.

### 4.2 Limitations

Several limitations should be acknowledged. First, the unequal group sizes, particularly in the CG, may limit the generalizability of the findings. Second, the study focused exclusively on amateur female players, and results may not be directly transferable to male or elite populations. Third, while biomechanical and performance outcomes were evaluated, injury incidence was not tracked, and long-term effects remain unknown. Finally, kinematic and kinetic analyses relied on markerless motion capture technology. While this method allows for efficient and non-invasive data collection in field settings, it might be less precise than marker-based systems, particularly in estimating very small joint angles and moments. Consequently, some knee kinetics and kinematics outcomes, especially subtle differences between limbs should be interpreted with caution. Future studies should aim to include larger cohorts, assess longitudinal outcomes, evaluate effects on injury rates, and combine markerless and gold-standard marker-based to further validate the Football+ program.

## Ethics approval and consent to participate

This study received ethics approval from Ethics committee of TU Dortmund University.

## Consent for publication

The authors have the same contribution, read, and approved the final manuscript.

## Availability of data and material

The datasets used during the current study are available from the corresponding author on reasonable request.

## Trial registration number

DRKS00036644.

## Authors' contributions

MA derived the study concept, developed the Football+ program, and drafted the manuscript. MH reviewed and revised the manuscript and refined the methodology. BT, MS^1^, performed the interventions, data collection, and preparation. MS^2^ managed the intervention phase and performed the introductory workshops, KN, performed the statistical analysis, TJ supervised the project, reviewed, and refined the manuscript.

∗ The manuscript is **not** already available on a preprint server.

## Funding declaration

No sources of funding were used to assist in the preparation of this article.

## Declaration of competing interest

None.

## References

[1] Sople D., Wilcox R.B. (2025). Dynamic warm-ups play pivotal role in athletic performance and injury prevention. Arthroscopy, Sports Medicine, and Rehabilitation.

[2] Asgari M, Nazari B, Bizzini M, Jaitner T (2023). Effects of the FIFA 11+ program on performance, biomechanical measures, and physiological responses: A systematic review. J Sport Health Sci.

[3] Zarei M. (2018). Long-term effects of the 11+ warm-up injury prevention programme on physical performance in adolescent male football players: a cluster-randomised controlled trial. J Sports Sci.

[4] Impellizzeri F.M. (2013). Physiological and performance responses to the FIFA 11+ (part 2): a randomised controlled trial on the training effects. J Sports Sci.

[5] Winstanley C., Reid D., Fulcher M.L. (2023). Suggested improvements to the 11+ as identified by coaches, players, strength and conditioning staff and medical staff in New Zealand football. BMJ Open Sport & Exercise Medicine.

[6] Donaldson A. (2019). A concept mapping approach to identifying the barriers to implementing an evidence-based sports injury prevention programme. Inj Prev.

[7] O'Brien J., Finch C.F. (2016). Injury prevention exercise programmes in professional youth soccer: understanding the perceptions of programme deliverers. BMJ open sport & exercise medicine.

[8] Wilke J. (2018). Is the message getting through? Awareness and use of the 11+ injury prevention programme in amateur level football clubs. PLoS One.

[9] Al Attar W.S.A. (2017). Implementation of an evidence-based injury prevention program in professional and semi-professional soccer. Int J Sports Sci Coach.

[10] Sadigursky D. (2017). The FIFA 11+ injury prevention program for soccer players: a systematic review. BMC Sports Sci Med Rehabil.

[11] Owoeye O.B.A., VanderWey M.J., Pike I. (2020). Reducing injuries in soccer (football): an umbrella review of best evidence across the epidemiological framework for prevention. Sports Med Open.

[12] Al Attar W.S.A. (2016). How effective are F-MARC injury prevention programs for soccer players? A systematic review and meta-analysis. Sports Med.

[13] Bizzini M., Dvorak J. (2015). Fifa 11+: an effective programme to prevent football injuries in various player groups worldwide—a narrative review. Br J Sports Med.

[14] Thorborg K. (2017). Effect of specific exercise-based football injury prevention programmes on the overall injury rate in football: a systematic review and meta-analysis of the FIFA 11 and 11+ programmes. Br J Sports Med.

[15] Al Attar W.S.A. (2025). Effectiveness of the FIFA 11+ injury prevention programs in reducing acute lower back injury among soccer players: A systematic review and meta-analysis. Int J Sports Sci Coach.

[16] O'Brien J., Young W., Finch C. (2017). The use and modification of injury prevention exercises by professional youth soccer teams. Scand J Med Sci Sports.

[17] Asgari M. (2022). Effects of the FIFA 11+ and a modified warm-up programme on injury prevention and performance improvement among youth male football players. PLoS One.

[18] O'Brien J., Young W., Finch C.F. (2017). The delivery of injury prevention exercise programmes in professional youth soccer: comparison to the FIFA 11+. J Sci Med Sport.

[19] Bathe C. (2023). Training interventions to reduce the risk of injury to the lower extremity joints during landing movements in adult athletes: a systematic review and meta-analysis. BMJ Open Sport & Exercise Medicine.

[20] Dalen-Lorentsen T., O'Brien J., Harøy J. (2024). Real-world implementation of the copenhagen adduction exercise: what do football teams modify and why?. BMJ Open Sport & Exercise Medicine.

[21] Grooms D.R. (2024). Neurocognitive & ecological motor learning considerations for the 11+ ACL injury prevention program: a commentary. Int J Sports Phys Ther.

[22] Asgari M. (2023). Acute effects of the FIFA11+ and football+ warm-ups on motor performance. A crossover randomized controlled trial. PLoS One.

[23] Asgari M. (2026). Four months training with the Football+ and 11+ improves the landing quality of female players. Int J Sports Med.

[24] O'Brien J. (2019). A new model for injury prevention in team sports: the Team-sport injury prevention (TIP) cycle. Science and Medicine in Football.

[25] Finch C. (2006). A new framework for research leading to sports injury prevention. J Sci Med Sport.

[26] Ramachandran A.K. (2025). Influence of Neuromuscular Training Interventions on Jump-Landing Biomechanics and Implications for ACL Injuries in Youth Females: A Systematic Review and Meta-analysis. Sports Med.

[27] Leppänen M. (2017). Stiff landings are associated with increased ACL injury risk in young female basketball and floorball players. Am J Sports Med.

[28] Mausehund L., Krosshaug T. (2024). Knee biomechanics during cutting maneuvers and secondary ACL injury risk: a prospective cohort study of knee biomechanics in 756 female elite handball and soccer players. Am J Sports Med.

[29] Dix C. (2021). Biomechanical changes during a 90° cut in collegiate female soccer players with participation in the 11. Int J Sports Phys Ther.

[30] Arundale A.J.H. (2018). Changes in biomechanical knee injury risk factors across two collegiate soccer seasons using the 11+ prevention program. Scand J Med Sci Sports.

[31] Bahari Fard R. (2022). Assessing changes in static and dynamic postural stability in youth football players following the FIFA 11+ injury prevention program. Sci Sports.

[32] Faul F. (2009). Statistical power analyses using G∗ power 3.1: tests for correlation and regression analyses. Behav Res Methods.

[33] Soligard T. (2008). Comprehensive warm-up programme to prevent injuries in young female footballers: cluster randomised controlled trial. Br Med J.

[34] Mok K.M., Bahr R., Krosshaug T. (2018). Reliability of lower limb biomechanics in two sport-specific sidestep cutting tasks. Sports Biomech.

[35] Ripic Z. (2023). A comparison of three-dimensional kinematics between markerless and marker-based motion capture in overground gait. J Biomech.

[36] Riazati S. (2022). Absolute reliability of gait parameters acquired with markerless motion capture in living domains. Front Hum Neurosci.

[37] Ali N., Rouhi G., Robertson G. (2013). Gender, vertical height and horizontal distance effects on single-leg landing kinematics: implications for risk of non-contact ACL injury. J Hum Kinet.

[38] Höner O., Roth K. (2015). Erläuterungen zu den individuellen Spielerauswer-tungen im Rahmen der technisch-motorischen Leistungsdiagnostik an den DFB-Stützpunkten. Zugriff am.

[39] Castagna C. (2013). Concurrent validity of vertical jump performance assessment systems. J Strength Condit Res.

[40] Rhea M.R. (2004). Determining the magnitude of treatment effects in strength training research through the use of the effect size. J Strength Condit Res.

[41] Blanca M.J. (2023). Non-normal data in repeated measures ANOVA: impact on type I error and power. Psicothema.

[42] Eirale C. (2013). Low injury rate strongly correlates with team success in Qatari professional football. Br J Sports Med.

[43] Krosshaug T. (2016). The vertical drop jump is a poor screening test for ACL injuries in female elite soccer and handball players: a prospective cohort study of 710 athletes. Am J Sports Med.

[44] Hewett T.E. (2005). Biomechanical measures of neuromuscular control and valgus loading of the knee predict anterior cruciate ligament injury risk in female athletes: a prospective study. Am J Sports Med.

[45] Myer G.D. (2013). The influence of age on the effectiveness of neuromuscular training to reduce anterior cruciate ligament injury in female athletes:a meta-analysis. Am J Sports Med.

[46] Benjaminse A. (2019). Revised approach to the role of fatigue in anterior cruciate ligament injury prevention: a systematic review with meta-analyses. Sports Med.

[47] Yu B., Garrett W.E. (2007). Mechanisms of non-contact ACL injuries. Br J Sports Med.

[48] Myer G.D. (2007). Differential neuromuscular training effects onACL injury risk factors in"high-risk" versus "low-risk" athletes. BMC Muscoskelet Disord.

[49] Markovic G., Mikulic P. (2010). Neuro-musculoskeletal and performance adaptations to lower-extremity plyometric training. Sports Med.

[50] Chaudhari A.M.W. (2020). Reducing core stability influences lower extremity biomechanics in novice runners. Med Sci Sports Exerc.

[51] Dello Iacono A., Johnny P., Ayalon M. (2016). Core stability training on lower limb balance strength. J Sports Sci.

[52] Chappell J.D., Limpisvasti O. (2008). Effect of a neuromuscular training program on the kinetics and kinematics of jumping tasks. Am J Sports Med.

[53] Herman D.C. (2022). Effect of strength training on jump-landing biomechanics in adolescent females. Sport Health.

[54] Delahunt E. (2007). Neuromuscular contributions to functional instability of the ankle joint. J Bodyw Mov Ther.

[55] Hübscher M. (2010). Neuromuscular training for sports injury prevention: a systematic review. Med Sci Sports Exerc.

[56] Behm D.G. (2016). Acute effects of muscle stretching on physical performance, range of motion, and injury incidence in healthy active individuals: a systematic review. Appl Physiol Nutr Metabol.

[57] Abade E. (2017). Effects of different re-warm up activities in football players' performance. PLoS One.

[58] Thomas K., French D., Hayes P.R. (2009). The effect of two plyometric training techniques on muscular power and agility in youth soccer players. J Strength Condit Res.

[59] Daneshjoo A. (2013). Effects of the 11+ and harmoknee warm-up programs on physical performance measures in professional soccer players. J Sports Sci Med.

[60] Zois J., Bishop D., Aughey R. (2015). High-intensity warm-ups: effects during subsequent intermittent exercise. Int J Sports Physiol Perform.

[61] Lopes M. (2019). Balance and proprioception responses to FIFA 11+ in amateur futsal players: short and long-term effects. J Sports Sci.

[62] Neil-Sztramko S.E. (2019). Updated systematic review of exercise studies in breast cancer survivors: attention to the principles of exercise training. Br J Sports Med.

[63] Whalan M. (2019). The incidence and burden of time loss injury in Australian men's sub-elite football (soccer): a single season prospective cohort study. J Sci Med Sport.

